# Genotype distribution and risk factors of *Toxoplasma gondii* infection in animals of Trishal, Bangladesh

**DOI:** 10.1371/journal.pone.0340911

**Published:** 2026-01-13

**Authors:** Pijush Kumar Biswas, Dipesh Aryal, AL Nur Tarak, Anisuzzaman, Md. Shahiduzzaman

**Affiliations:** Department of Parasitology, Faculty of Veterinary Science, Bangladesh Agricultural University, Mymensingh, Bangladesh; National Research Centre, EGYPT

## Abstract

*Toxoplasma gondii* is a globally significant parasite with no genotyping data available from Bangladesh. This study aimed to determine the genotype distribution and associated risk factors of *T. gondii* infection in animals in Trishal Upazila, Mymensingh district. From June 2020 to December 2023, a total of 170 samples from cattle, goats, sheep, cats, dogs, chickens, and rodents in the study area and examined using nested PCR targeting the B1 gene of *T. gondii*. Positive samples were genotyped by multilocus nested PCR-RFLP at ten genetic markers. Risk factor data were analyzed using logistic regression at the farm or owner level. The overall infection rate of 21.76% (37/170), with the highest prevalence observed in cyst samples from slaughtered cattle (42.9%) and cat feces (33.3%). Genotyping revealed a predominance of Type I and mixed genotypes (I/II, I/III) in cattle, cats, dogs, and rodents, while goats and sheep predominantly carried Type II or III. Chickens exhibited a mixed II/III allelic pattern with Type I alleles at the *C22-8* locus. The use of pond/river water as a drinking source was a significant risk factor, with animals from these sources showing more than threefold higher odds of infection compared to tube-well users (p = 0.038). Backyard or small-holder farming, improper carcasses/offals/ placenta disposal, lack of rodent control, and unhygienic slaughter practices showed positive but non-significant associations with infection. This first report of *T. gondii* genotyping in Bangladesh highlights the presence of diverse and potentially virulent genotypes in food-producing animals, posing a notable zoonotic risk.

## Introduction

*Toxoplasma gondii* is a ubiquitous, apicomplexan parasite of warm-blooded animals that can cause several clinical syndromes including encephalitis, chorioretinitis, congenital infection and neonatal mortality [1]. *T. gondii* is an important zoonotic pathogen and a major cause of reproductive failure associated with abortion in cow, sheep and goat. At present, it is considered as a major cause of abortion in goats worldwide [2]. Pregnancy losses due to embryonic mortality and abortion are the major constraints in completion of gestation in all livestock animals. These are major impediments in the reproductive as well as productive efficiency in livestock animals. It leads to huge economic losses to the farmers, in the form of expected new-born as well as delay in production time especially in dairy industry [3].

The parasite has a worldwide distribution and it is mainly transmitted by food or water contaminated with oocysts dispersed by cats and other felines (definitive hosts) [4]. Raw or under cooked meat containing tissue cysts or un-pasteurized milk containing tachyzoites, and trans-placental transmission also occurs [4]. Studies report that *T. gondii* infection is widespread in Bangladesh, affecting 16–39% of humans and 8–70% of domestic animals [5]. Seroprevalence studies have detected antibodies in cattle (16.10%), sheep (17.65%), and goats (12.09–32%) [6,7]. Additionally, 3.4% of rodents from food production and storage facilities tested positive for *T. gondii* DNA, indicating their role in transmission [8]. The high prevalence in meat-producing animals poses a significant risk to humans, especially pregnant women, as highlighted by a hospital survey in Mymensingh and Rangpur that found a 25.3% seroprevalence among women [9]. Undiagnosed cases of abortion, stillbirth, and retained placentas also contribute to livestock losses [10]. While abortion studies in livestock have been conducted internationally, no such investigations focusing on *T. gondii* have been carried out in either humans or animals in Bangladesh [11]. Furthermore, the genetic diversity and transmission routes of the parasite remain largely unexplored [5].

Understanding local strain diversity through genotyping is crucial, as it influences virulence, transmission dynamics, and clinical outcomes. Certain genotypes are linked with higher pathogenicity, congenital transmission, and severe disease in both humans and animals. Without genotypic data, assessing strain-specific public health risks or comparing local strains with global and regional genotypes it is not possible.

Despite *T. gondii* being extensively studied worldwide, genotype data from Bangladesh is lacking. This gap is particularly concerning given the country’s high density of livestock, frequent human–animal interactions, widespread contact with stray animals, and climatic conditions favourable for the survival of oocysts. In addition, low awareness of zoonotic diseases and the absence of molecular surveillance further increase the potential risk. Generating genotypic data would aid in designing targeted control measures, guiding vaccine development, and improving diagnostic accuracy for toxoplasmosis management in Bangladesh.

*T. gondii* exhibits notable genetic diversity, with three main clonal lineages Types I, II, and III dominating in North America and Europe, where Type I is highly virulent and often linked to congenital toxoplasmosis [12,13]. In contrast, Asia, especially China, reports both clonal types and unique strains such as ToxoDB#9 (Chinese I), reflecting broader genetic variation [14,15]. In South Asia, particularly India, recombinant and atypical genotypes, including Type II/III recombinants, have been detected in clinical cases [16], aligning with global patterns of higher diversity in tropical regions [17]. Despite high seroprevalence across South Asia [18] including Bangladesh [6,7], no genotype-level data is available in Bangladesh, highlighting the need for molecular characterization to better understand local strain diversity and its public health implications.

In this study, nested PCR (nPCR) targeting the multi-copy B1 gene was used for molecular detection of *T. gondii* due to its high sensitivity and reliability in identifying low parasite loads across host species [19, 20]. Multilocus PCR-RFLP genotyping was performed using ten genetic markers (SAG1, 5′SAG2, 3′SAG2, alt. SAG2, SAG3, BTUB, GRA6, C22-8, C29-2, and PK1), a cost effective and reproducible approach for classifying clonal and recombinant lineages in resource limited settings [21–23]. This method has been widely applied globally, enabling comparative analysis with international genotype databases while providing sufficient resolution for population-level studies [21].

The shared environments such as farms, slaughterhouses, and surrounding fields were selected because they represent interfaces where multiple animal species and humans interact, facilitating potential cross-species transmission of *T. gondii*. Studying these environments offers insights into epidemiological links and transmission pathways critical for effective zoonotic disease control. Therefore, the primary objective of this study was to genetically characterize *T. gondii* isolates from multiple animal species in shared environments of Trishal, Bangladesh, using multilocus PCR-RFLP. The secondary objective was to identify potential risk factors associated with infection and to provide, molecular insights into the genetic makeup and zoonotic implications of *T. gondii* circulating in the region.

## Materials and methods

### Study site and sample collection

The study was conducted in Trishal Upazila, located within the Mymensingh district of Bangladesh (24°28’N – 24°40’N, 90°18’E – 90°30’E). The area was selected because it represents a mixed agro-ecological environment with both organized (military dairy) and smallholder livestock farms, as well as open slaughterhouses and peri-domestic animal settings that may facilitate *Toxoplasma gondii* transmission.

The samples were collected from different villages of unions including Trishal, Rampur, Kanihari, Kanthal, Bailar, Dhanikhola, Mathbari, Sakhua, Harirampur, Amoabari, Mokshapur, and Bali Para. From June 2020 to December 2023, a total of 170 biological samples were collected from multiple animal species across 84 farms/households and 6 slaughterhouses (Table 1). All samples were collected following a stratified convenience approach to ensure representation across different animal species, management systems, and environmental settings within the upazila. Sampling included aborted fetal tissues, placentas, hearts, feces, brains, and cysts, depending on host species. Aborted fetal tissues (heart) were collected from 27 cattle (Holstein Friesian and indigenous breeds), 15 Black Bengal goats (*Capra hircus*), and 12 sheep (*Ovis aries*), along with 16 placental tissues from aborted cows. Additionally, 37 heart tissues and 7 cyst samples were collected from slaughtered cattle. Faecal samples were obtained from 18 cats (*Felis catus*) and 13 dogs (*Canis lupus familiaris*) roaming near slaughterhouses. Brain tissues from 14 native chickens (*Gallus gallus domesticus*) were collected from free-ranging village birds. Eleven rodents (5 *Bandicota bengalensis*, 6 *Rattus rattus*) were captured from rice fields adjacent to slaughter areas, and their brain tissues were sampled. All samples were aseptically collected using sterile instruments, transported to the laboratory in ice-cooled containers, and stored at –20°C. Tissue samples were preserved in 98% ethanol for up to one week prior to molecular analysis.

**Table 1:** Distribution of collected samples (n = 170) by host species, breed, source, and sampling location.

Host Species	Breed/ Scientific Name	Sample Type(s)	Source/ Location	Number of Samples	Sample code
**Cattle**	Holstein Friesian	Aborted fetal tissue (heart)	Military Dairy Farm	7	A
	Holstein Friesian	Aborted fetal tissue (heart)	Local village dairy farm	9	B
	Local breed (*Bos indicus*)	Aborted fetal tissue (heart)	Farmers’ houses	11	C
	Mixed breed (slaughtered sterile cow)	Heart tissue	Open slaughterhouses near live markets	37	D
	Mixed breed (slaughtered sterile cow)	Cyst samples	Open slaughterhouses near live markets	7	E
	Holstein Friesian	Placental tissue	Aborted cows, farms	16	F
**Goat**	Black Bengal (*Capra hircus*)	Aborted fetal tissue (heart)	Rural farms and households	15	G
**Sheep**	*Ovis aries*	Aborted fetal tissue (heart)	Rural farms and households	12	H
**Chicken**	Deshi native (*Gallus gallus domesticus*)	Brain tissue	Purchased from village free-ranging system	14	I
**Cat**	Street/stray *Felis catus*	Fecal samples	Around slaughterhouses and market peripheries	18	J
**Dog**	Street/stray *Canis lupus familiaris*	Fecal samples	Around slaughterhouses and market peripheries	13	K
**Rodent**	*Bandicota bengalensis*, *Rattus rattus*	Brain	Rice fields adjacent to slaughterhouse areas	11	L

Total number of samples collected: 170. Total farms 84 (40 cattle farms, 18 goat farms, 12 sheep farms, 14 chicken household) and 6 slaughter houses, each code (A–L) represents one genotyped isolate.

A total of 84 epidemiological units were surveyed for risk factor analysis. A farm was classified as positive if at least one sample from that farm tested positive for *T. gondii* DNA by PCR. Thus, prevalence and genotyping analyses were conducted at the sample level (n = 170), whereas risk factor analysis was conducted only at the farm level (n = 84). Variables exclusive to slaughterhouses were analyzed descriptively but not included in the logistic regression due to small subgroup size.

### Tissue digestion and DNA extraction

DNA extraction was performed on a range of biological samples, including aborted fetal heart tissue, mature heart tissue, brain tissue, placental tissue, cyst samples, and fecal specimens. Tissue digestion protocols were adapted according to sample type to optimize yield and purity.

For aborted fetal tissues and other soft tissues (heart, brain, placenta), enzymatic digestion was carried out using a modified trypsin-based protocol as described by [24,25]. Approximately 20 g of tissue was incubated in 50 mL of digestion solution containing trypsin 1:250 (2.5 g/L in phosphate-buffered saline, PBS) at 37 °C for 16 hours with continuous stirring. Following digestion, the suspension was centrifuged at 7000 rpm for 5 minutes. The resulting pellet was resuspended in 5 mL PBS and centrifuged again at 5000 rpm for 3 minutes to remove residual debris. The final pellet was resuspended in 5 mL PBS, and 300 µL of this homogenate was used for DNA extraction.

For cyst samples, mechanical disruption was performed prior to enzymatic digestion to ensure adequate lysis. Fecal samples were processed using direct lysis protocols optimized for stool matrices, including pre-treatment with lysis buffer and mechanical agitation to enhance oocyst rupture.

Genomic DNA from all sample types was extracted using the Monarch Genomic DNA Purification Kit (New England BioLabs Inc., USA), following the manufacturer’s protocol. DNA concentration and purity were assessed using spectrophotometry (NanoDrop™), and extracts were stored at −20 °C until downstream molecular analysis.

### PCR amplification of *Toxoplasma gondii* B1 Gene

Nested PCR targeting the *Toxoplasma gondii* B1 gene was performed to detect parasite DNA [22]. The primary PCR employed the primer pair JW 63 F (5′-GCACCTTTCGGACCTCAACAACCG-3′) and JW 62 R (5′-TTCTCGCCTCATTTCTGGGTCTAC-3′), which amplified a 288 bp fragment [26]. Each 25 µL reaction mixture contained 12.5 µL of GoTaq® Green Master mix (Promega, USA), 1.5 µL of each primer (10 pmol), 2 µL of template DNA, and 7.5 µL of nuclease-free water. Thermal cycling was performed in a MiniPCR thermocycler (Oxford, UK) under the following conditions: initial denaturation at 94 °C for 30 seconds; 35 cycles of denaturation at 94 °C for 15 seconds, annealing at 45 °C for 30 seconds, and extension at 72 °C for 45 seconds; followed by a final extension at 72 °C for 10 minutes.

The nested PCR used primers B22m F (5′-AACGGGCGAGTAGCACCTGAGGAGA-3′) and B23m R (5′-TGGGTCTACGTCGATGGCATGACAAC-3′), targeting a 114 bp fragment [27]. Each 25 µL reaction included 12.5 µL GoTaq® Green Master Mix, 1.5 µL of each nested primer (10 pmol), 0.5 µL of primary PCR product, and 9 µL of nuclease-free water. Cycling conditions for the nested PCR were identical to the primary reaction, except the number of cycles was reduced to 30.

Each PCR run included appropriate controls to ensure reliability. A confirmed *T. gondii*-positive DNA sample was used as a positive control, while nuclease-free water served as the negative control. An extraction blank, consisting of reagent-only processed alongside samples, was included to monitor for contamination. PCR products were resolved on 1.5% agarose gels stained with ethidium bromide and run in 1X TAE buffer. DNA bands were visualized under ultraviolet (UV) transillumination using a gel documentation system, and images were digitally captured for analysis.

### Genotyping of *Toxoplasma gondii*

Genotyping of *T. gondii* isolates was performed using a multilocus nested PCR-restriction fragment length polymorphism (PCR-RFLP) technique targeting 10 independent genetic markers [19,21]: SAG1, SAG2 (5′-SAG2 and 3′-SAG2), alt-SAG2, SAG3, BTUB, GRA6, C22-8, C29-2, PK1. For each marker, individual nested PCR assays were conducted in two amplification rounds (Table 2). The B1 gene–positive samples were subjected to multilocus PCR-RFLP genotyping. Samples that failed to amplify at one or more loci after repeated attempts were classified as partially genotyped. Only samples successfully amplified at all ten loci were considered fully genotyped.

**Table 2:** List of markers and corresponding nPCR product size, restriction enzymes, incubation temperature and time used in molecular characterization of *Toxoplasma gondii* strains obtained from representative samples.

Markers	Nested PCR Product (bp)	Restriction Enzymes	Incubation Temp and time	Reference
**SAG1**	390	Sau96I and HaeII	37 °C 1 h	[28]
**5′-SAG2**	242	MboI,	37 °C 1 h	[29]
**3′-SAG2**	222	HhaI		
**alt. SAG2**	546	HinfI and TaqI	37°C 30 min, 65°C 30 min	[12,30]
**SAG3**	311	Nci I	37°C 1 h	[31]
**BTUB**	411	BsiE I,Taq I	60 °C 1 h	[12,30]
**GRA6**	344	Mse I	37 °C 1 h	[30,32]
**C22-8**	521	BsmAI and MboII	37 °C 30 min, 55 °C 30 min	[12,30]
**C29-2**	446	HpyCH4IV and RsaI	37 °C 1 h	[12,30]
**PK1**	903	AvaI and RsaI	37 °C 1	[12,30]

The primary PCR for each locus was carried out in a 25 μL reaction mixture containing 100 ng of genomic DNA, 1 × PCR GoTaq® Green Master mix (Promega, USA) and 0.5 μM of each external primer specific to the respective marker. Thermal cycling conditions included an initial denaturation at 94 °C for 5 minutes, followed by 35 cycles of denaturation at 94 °C for 30 seconds, annealing at 60 °C for 30 seconds, and extension at 72 °C for 30 seconds, with a final extension at 72 °C for 5 minutes.

Nested PCR was performed using 1 μL of the primary PCR product as the template in a 25 μL reaction containing 1 × PCR GoTaq® Green Master mix (Promega, USA) and 0.3 μM of each internal primer. The thermal profile for nested PCR included an initial denaturation at 94 °C for 5 minutes, followed by 35 cycles of denaturation at 94 °C for 30 seconds, annealing at 60 °C for 1 minute, and extension at 72 °C for 1.5 minutes, with a final extension step at 72 °C for 10 minutes.

The amplified products from each marker were individually digested with specific restriction endonucleases (New England Biolabs, UK) based on the protocol established for each locus. Digested products were resolved on 2.5% agarose gels stained with ethidium bromide and visualized under UV transillumination. The restriction fragment patterns were analyzed to determine the genotype of each isolate by comparing the banding profiles to reference strains for genotype I, II, and III. For quality control, *T. gondii* RH strain (isolated from field and confirmed by sequencing) was included as a positive control in each PCR run.

### Statistical analysis

A cross-sectional survey was conducted across the 84 farms/households and 6 slaughterhouses to identify potential management and environmental factors associated with *T. gondii* infection in animals.

Data were collected using a structured questionnaire administered to farm owners, caretakers, or slaughterhouse personnel during on-site visits. The questionnaire, developed in English and translated into Bengali for field implementation, included both closed and open-ended questions covering farm type (commercial or backyard/ smallholder), cat exposure (presence of domestic or stray cats and their access to feed, housing, and birthing areas), rodent control practices in farm, farm waste management, and water source (tube well, pond, or river).

Information on reproductive history (abortion, stillbirths, and neonatal deaths within the past 12 months) and disposal practices for carcasses, offals, and placental tissues (burial, open field, or water body) was also collected to assess possible pathways of *T. gondii* transmission.

Information on slaughterhouse conditions, including hygiene, disinfection routines, use of protective clothing, and visitor access control, was collected from six slaughter facilities. Because these variables were not comparable to farm-level management factors, slaughterhouse data were analyzed descriptively rather than included in the logistic regression.

All collected data were coded and entered into Microsoft Excel and subsequently analyzed using IBM SPSS Statistics version 26.0 (IBM Corp., Armonk, NY, USA). Categorical variables were summarized as frequencies and percentages. Associations between potential risk factors and *Toxoplasma gondii* infection status were initially evaluated using chi-square (χ²) tests. Variables with *p* < 0.20 in the univariate analysis were then entered into a multivariable logistic regression model to identify independent predictors of infection. Adjusted odds ratios (aOR) and 95% confidence intervals (CI) were estimated using a backward stepwise likelihood ratio method. Model adequacy was evaluated using the Hosmer–Lemeshow goodness-of-fit test and Nagelkerke R^2^.

### Ethics statement

All sampling procedures were conducted with informed consent and under ethical approval from the Animal Welfare and Experimentation Ethics Committee (AWEEC) of Bangladesh Agricultural University, Mymensingh, under approval number AWEEC/BAU/2020(30). Informed consent was obtained from all animal owners and farm authorities prior to sample collection. The objectives of the study, sample collection procedures, and confidentiality of data were explained in the local language (Bangla). Aborted fetal tissues were collected following spontaneous abortion events reported by owners, with no intervention to induce abortion. Samples from slaughtered sterile cows were obtained post-mortem from routine abattoir processing. Sampling of stray cats, dogs, and rodents (having no authority) in the surrounding areas was carried out under the oversight of the local livestock authority. Tissue, samples from aborted fetus, chicken and rodents were isolated under approved field protocols. The ethics committee did not require written consent because no human biological materials were collected, and sampling procedures were non-invasive for the animals.

### Field site permission

Fieldwork and sample collection were conducted with the prior approval and oversight of the Upazila Livestock Officer, Trishal, under the Department of Livestock Services (DLS), Ministry of Fisheries and Livestock, Government of the People’s Republic of Bangladesh. Permission for sample collection at the Military Farm, Trishal, was formally obtained from the Commanding Officer of the Military Farm. Access to privately owned farms was granted by the respective owners after obtaining their informed verbal consent.

No additional government or environmental permits were required, as the study involved non-invasive collection of tissue and fecal samples from domesticated animals under veterinary supervision and in accordance with institutional animal welfare and ethical guidelines.

## Results

### PCR detection of *Toxoplama gondii*

**Fig 1:**
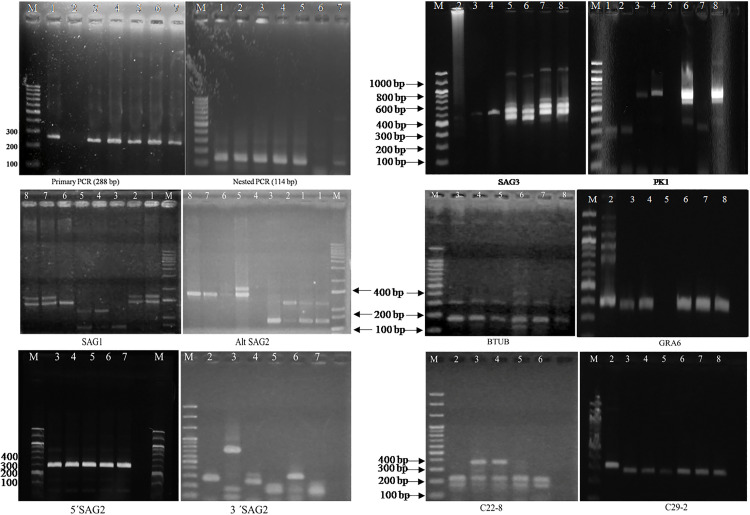
Detection and genotyping of *Toxoplasma gondii* using nested PCR and multilocus PCR-RFLP. Primary PCR gel image: M (Ladder-1kbp), Lane 1 = Positive control (*T. gondii* RH), 2 (Negative control), 3 = Cattle, 4 = Goat, 5 = Sheep, 6 = Chicken, 7 = Cat. nPCR gel image: 1 = Cattle, 2 = Goat, 3 = Sheep, 4 = Chicken, 5 = Cat, 6 = Negative control, 7 = Positive control (*T. gondii* RH (Type 1). Multilocus Nested PCR-RFLP analysis of *T. gondii* with 10 different genetic markers. Lane M represents molecular marker, lane 1 is *T. gondii* RH (Type I) strain (Reference), and 2-8 are samples (2 = Cattle, 3 = Goat, 4 = Sheep, 5 = Chicken, 6 = Cat, 7 = Dog, 8 = Rat). All the nested PCR products were digested with the restriction enzymes as given in Table 2 and the digested products were separated in 2.5% agarose gel.

PCR-based detection of *T. gondii* DNA was performed on 170 samples collected from a variety of host species, including cattle, goats, sheep, chickens, cats, dogs, and rodents in Trishal Upazila, Mymensingh, Bangladesh. Out of total samples 37 were found positive by nPCR (Fig 1). The highest prevalence was observed in cyst samples from slaughtered cattle (42.9%), indicating chronic infection and potential persistence of tissue cysts in meat animals (Table 3). Fecal samples from cats showed 33.3% infection, reflecting their role as definitive hosts and active environmental shedders of oocysts. Moderate infection was found in aborted fetal tissues (22.2%) and placental samples (26.7%) from ruminants supporting the possibility of transplacental transmission. Detection in brain tissues of free-ranging chickens (14.3%) and rodents (27.3%) suggests the involvement of both sylvatic and domestic transmission cycles. These findings highlight the widespread presence and genetic diversity of *T. gondii* across multiple host species and sample types, underscoring the zoonotic risk posed by contaminated environments and undercooked animal products.

**Table 3:** PCR-based detection of *Toxoplasma gondii* in various host samples.

Host Species	Sample Type	Number Tested	Number Positive	Positivity Rate (%)
**Cattle**	Aborted fetal tissue (heart)	27	6	22.2%
	Placental tissue	16	4	25.0%
	Slaughtered sterile cow tissue (heart)	37	5	13.5%
	Slaughtered sterile cow tissue (cyst)	7	3	42.9%
**Goat**	Aborted fetal tissue (heart)	15	4	26.7%
**Sheep**	Aborted fetal tissue (heart)	12	3	25.0%
**Chicken**	Brain tissue	14	2	14.3%
**Cat**	Fecal samples	18	6	33.3%
**Dog**	Fecal samples	13	1	7.7%
**Rodent**	Brain tissue	11	3	27.3%
	Total	170	37	21.76%

### Genotyping

Multilocus PCR-RFLP genotyping of *T. gondii* was successfully performed on the positive samples confirmed by nPCR (S1 Raw Images). Genotypic analysis using ten genetic markers (SAG1, SAG2, alt. SAG2, SAG3, BTUB, GRA6, C22-8, C29-2, and PK1) revealed a predominance of Type I and mixed genotypes (I/II, I/III) among isolates from cattle, cats, dogs, and rodents (Fig 1 & Table 4). In contrast, goats and sheep more frequently harboured Type II and III genotypes, whereas chickens exhibited a mixed II/III allelic pattern with Type I alleles at the C22-8 locus.

**Table 4:** Multilocus PCR-RFLP genotyping for *Toxoplasma gondii* isolates from different host species.

Samples codes (Host species)	Genetic Markers							
	SAG1	alt. SAG2	SAG3	BTUB	GRA6	C22-8	C29-2	PK1
**RH (Type I)**	I	I	I	I	I	I	I	I
**A -F (Cattle)**	I/III	I	I	–	I	I	I	I
**G (Goat)**	II/III	III	II	II	II	II	II	II
**H (Sheep)**	II	–	II	II	II	II	II	II
**I (Chicken)**	II/III	II	II	II	–	I	II	–
**J (Cat)**	I/II	I	II	II	II	I	I	II
**K (Dog)**	I	I	II	II	II	–	I	II
**L (Rodent)**	I	I	I	II	II	–	I	II

Each code (A–L) represents a single *T. gondii*-positive isolate from the indicated host species. The RH strain (Type I) was used as a reference control. (–) indicates samples that failed to amplify at one or more loci after repeated attempts (partially genotyped).

Cat-derived isolates exhibited allelic combinations suggestive of mixed or recombinant genotypes rather than multiple isolates, reflecting the cat’s role as the definitive host and active oocyst shedder. Each code (A–L) represents one genotyped isolate. Mixed genotypes indicate the presence of alleles from more than one clonal lineage within a single isolate, reflecting either co-infection with multiple strains or recombination events.

Notably, mixed or recombinant genotypes were detected across multiple species, suggesting environmental circulation of non-clonal strains and potential recombination events. These findings underscore the genetic heterogeneity of *T. gondii* in domestic and commensal animals in Bangladesh and highlight the public health implications of virulent and atypical genotypes circulating in food-producing animals.

### Risk factors analysis

#### Slaughtering practices and environmental contamination.

**Fig 2:**
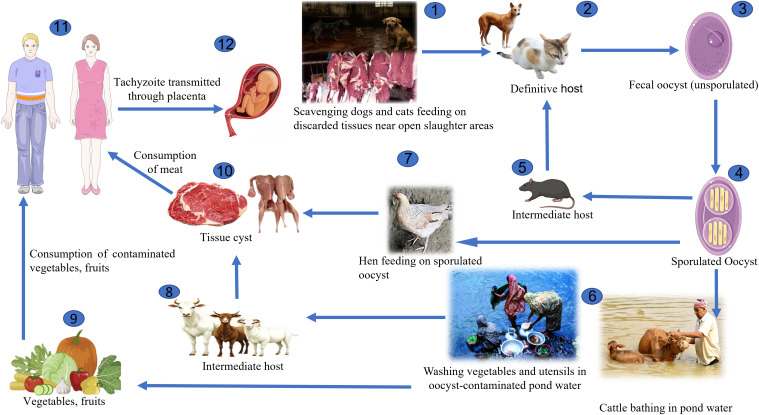
Possible transmission pathways of *Toxoplasma gondii* in the shared environment of Trishal, Mymensingh district. (1) scavenging dogs and cats feeding on discarded tissues near open slaughter areas; (2) definitive host (cats and other felines); (3) faecal oocyst (unsporulated); (4) sporulated oocyst; (5) intermediate host (e.g., rodent); (6) cattle bathing in pond water or washing vegetables and utensils in oocyst-contaminated pond water; (7) hens feeding on sporulated oocysts; (8) intermediate host livestock (e.g., cattle, goat, sheep); (9) vegetables and fruits; (10) tissue cysts in meat; (11) human consumption of contaminated meat, vegetables, and fruits; (12) tachyzoite transmission through placenta (congenital transmission).

In Trishal cattle, goats and sheep are slaughtered in a live open market without strict adherence to hygienic practices. This situation presents a significant risk factor for environmental contamination and zoonotic transmission of *T. gondii*. Discarded raw meat, cyst-containing muscles and offal are freely dumped in open spaces where infected material containing *T. gondii* tissue cysts is scavenged by free-ranging cats, rats and dogs. The current study detected *T. gondii* DNA in various host species (Table 3), reinforcing the concern for ongoing transmission cycles in this environment (Fig 2).

In the study areas, cats and dogs frequently defecate in homestead gardens, farms, and agricultural lands, creating a significant source of environmental contamination with *T. gondii* oocysts. These areas are commonly accessed by farmers and female agricultural workers, who may come into direct contact with contaminated soil during gardening or farming activities. Rainwater runoff and surface drainage can carry oocysts from contaminated soil or feces into nearby ponds and water bodies**,** which serve as sources of water for both human use and livestock consumption (Fig 2). This poses a risk of infection to people bathing, washing, or using the water for domestic purposes, as well as to animals that drink from these contaminated sources.

In addition, many farmers particularly those with limited education wash freshly harvested vegetables in pond water to remove dirt and soil before taking them to local markets. These vegetables, if contaminated with oocysts, can enter the food chain without further washing or cooking. Shoppers may purchase such produce from markets and bring it into the household, where handling contaminated raw vegetables in the kitchen can expose food handlers, family members, and others to *T. gondii* via contaminated hands, surfaces, or utensils. This farm-to-market contamination pathway underlines a critical public health risk and highlights the importance of improving water safety, food hygiene, and public awareness, especially in rural communities.

#### Role of scavenging cats, dogs, and rats.

Scavenging animals particularly cats, dogs, and rodents are drawn to the discarded meat and offals in the study areas (Fig 2). Cats, the definitive host of *T. gondii*, become infected by ingesting tissue cysts from contaminated offals or rodents. In this study, 33.3% of cat fecal samples were PCR-positive, indicating active oocyst shedding, which can contaminate surrounding soil and water.

Rodents feeding on infected tissues or environments act as intermediate hosts. 27.3% of tested rodent brain samples were positive for *T. gondii*, showing their involvement in the parasite’s life cycle and serving as prey for cats, thereby perpetuating the transmission loop (Table 3). Dogs become infected by ingesting the infected tissue cyst or offals later excreting oocyst in environment. In this study, 7.7% of dog fecal samples tested positive, supporting their potential role in *T. gondii* dispersal.

This likely represents the transmission cycle of *T. gondii* infection in Trishal accompanied by unhygienic handling at live animal markets, the presence of scavenging cats and dogs, the low level of awareness among local people about the health effects of the infection, and the wide spread of the contamination of the environment (Fig 2). These factors are interrelated and maintain an enduring cycle of transmission **a**mong animals, the environment, and human beings, thereby posing a constant threat to public health.

#### Undiagnosed abortion and lack of knowledge.

In livestock (cattle, goats, sheep) of Bangladesh, abortions in animals are rarely investigated for underlying infectious causes. Farmers typically dispose aborted fetuses, placentas, and fluids in open fields or ponds, without proper biosecurity measures. If the abortion is caused by *T. gondii*, these materials may contain infectious tissue cysts or oocysts, which remain viable in the environment and pose a direct risk to scavenging cats, dogs, rats, and eventually humans. This contributes to the maintenance of the transmission cycle, especially because cats become infected by consuming aborted tissues, then shed millions of oocysts in the environment.

In Bangladesh, early pregnancy loss or stillbirth in women is rarely medically investigated, and *T. gondii* is seldom considered a potential causative agent. As a result, toxoplasmosis-related pregnancy complications including congenital toxoplasmosis are likely underreported and frequently go unrecognized. This is largely due to a lack of diagnostic facilities, limited awareness among healthcare providers and the public, and the absence of routine antenatal screening. Infected women, often unaware of their condition, may continue daily household activities, potentially contaminating food, water, and domestic surfaces, particularly where hygiene practices are inadequate. Such scenarios can contribute to household-level transmission of *T. gondii*, posing risks to other family members, including children and immunocompromised individuals.

In the multivariable logistic regression model (Table 5), animals from farms using pond or river water had significantly higher odds of *T. gondii* infection (aOR = 3.25, 95% CI: 1.02–10.37, p = 0.046). Although not statistically significant, farms without rodent control (aOR = 3.72, p = 0.066) and those with abortion history (aOR = 2.81, p = 0.068) suggested a potential association with *T. gondii* infection.

**Table 5:** Multivariable logistic regression analysis of potential risk factors associated with *Toxoplasma gondii* infection in livestock (n = 84).

Variable	Category	Adjusted OR (aOR)	95% CI	p-value
**Farm type**	Commercial	1 (Ref)	0.84–10.46	0.089
	Backyard/Smallholder	2.97		
**Cat presence on farm**	No	1 (Ref)	0.71–9.94	0.142
	Yes	2.65		
**Rodent control implemented**	Yes	1 (Ref)	0.92–15.10	0.066
	No	3.72		
**Source of water**	Tube well	1 (Ref)	1.02–10.37	0.046*
	Pond/River	3.25		
**Abortion history (past 12 months)**	No	1 (Ref)	0.93–8.47	0.068
	Yes	2.81		

Logistic regression analysis was performed using data from farm/household units only (n = 84). Variables with *p* < 0.20 in the univariate analysis were included in the multivariable logistic regression model. OR = Odds Ratio; CI = Confidence Interval. Model fit: Hosmer–Lemeshow χ² = 6.18, df = 8, p = 0.63; Nagelkerke R^2^ = 0.29. * indicates statistically significant association (*p* < 0.05).

Farm type and cat presence on farm were not independently associated with infection after adjustment. These findings suggest that contaminated surface water may be the key route of *T. gondii* transmission, while other management and biosecurity factors may contribute to risk in smaller-scale operations.

Out of six slaughterhouses examined, two (33.3%) were PCR positive for *T. gondii*. Positivity was observed only in facilities lacking regular disinfection, consistent use of protective clothing, and visitor access control (Table 6).

**Table 6:** Descriptive characteristics of slaughterhouse environments surveyed for *Toxoplasma gondii* contamination (n = 6).

Variable	Category	No. of slaughterhouses (%)	*T. gondii* Positive n (%)
**Hygienic condition**	Hygienic/ Controlled	2 (33.3%)	0 (0%)
	Poor hygiene/ Uncontrolled	4 (66.7%)	2 (33.3%)
**Disinfection routine**	Regular (daily/ weekly)	3 (50.0%)	0 (0%)
	Irregular/ None	3 (50.0%)	2 (33.3%)
**Use of protective clothing**	Consistent	2 (33.3%)	0 (0%)
	Inconsistent/ None	4 (66.7%)	2 (33.3%)
**Visitor access control**	Restricted	1 (16.7%)	0 (0%)
	Unrestricted	5 (83.3%)	2 (33.3%)
**Total**		6 (100%)	2 (33.3%)

Data presented descriptively due to small sample size (n = 6); no inferential statistics performed. Two slaughterhouses (33.3%) tested positive for *T. gondii* DNA by PCR. “Hygienic/Controlled” = routine cleaning, disinfectant use, drainage, and access control observed. “Poor hygiene/Uncontrolled” = no systematic cleaning, visible waste accumulation, and free animal access.

## Discussion

*Toxoplasma gondii* is a globally important zoonotic parasite with significant implications for both animal production and public health. In Bangladesh, several serological surveys have demonstrated its endemic presence in livestock and humans, with reported infection rates of 12% in cattle, 32% in goats, and 40% in sheep, and human seroprevalence ranging from 16% to 39% [5,7]. These studies highlight the persistent exposure risk within rural and peri-urban communities, where close contact between humans, animals, and contaminated environments facilitates transmission. Within this context, the present study extends existing knowledge by providing molecular evidence of *T. gondii* circulation and genetic diversity among multiple animal hosts in Trishal Upazila, offering a new dimension to the understanding of its epidemiology in Bangladesh.

Globally, the parasite exhibits a predominantly clonal population structure, with three major lineages Type I, II, and III accounting for most infections in Europe and North America. However, atypical and recombinant genotypes are increasingly reported in South America, Africa, and parts of Asia, suggesting regional variation in strain virulence and transmission dynamics [33,34]. Understanding the genetic makeup of local isolates is essential for assessing zoonotic risk and guiding public health interventions. This study provides the molecular characterization of *T. gondii* isolates from multiple animal hosts in Trishal Upazila, offering new insights into the parasite’s genetic landscape and its potential implications for zoonotic transmission in Bangladesh.

The detection of *T. gondii* DNA in 21.8% (37/170) of samples collected from diverse host species in Trishal Upazila underscores the parasite’s widespread circulation in both domestic and peri-domestic ecosystems. The highest prevalence was observed in tissue cysts from slaughtered cattle (42.9%), suggesting chronic infection and the persistence of bradyzoites in edible tissues. This finding aligns with previous reports from Bangladesh and other endemic regions, where cattle are frequently exposed to environmental oocysts but often remain asymptomatic carriers [7,35]. The presence of tissue cysts in meat animals raises concerns about foodborne transmission, particularly in communities where consumption of undercooked beef is common.

Fecal samples from cats showed a notable infection rate of 33.3%, reaffirming their role as definitive hosts responsible for shedding environmentally resistant oocysts. Cats are central to the transmission cycle of *T. gondii*, and their proximity to livestock and human dwellings in rural Bangladesh facilitates widespread environmental contamination [36]. The detection of *T. gondii* in aborted fetal tissues (22.2%) and placental sample (25.0%) from ruminants further supports the possibility of vertical transmission, which has been documented in both experimental and field settings [37]. These findings are particularly relevant for livestock productivity and reproductive health, as congenital toxoplasmosis can lead to abortion, stillbirth, and neonatal mortality.

The identification of *T. gondii* DNA in brain tissues of free-ranging chickens (14.3%) and rodents (27.3%) suggests active transmission within both sylvatic and domestic cycles. Chickens, often considered sentinels for environmental contamination, acquire infection through ingestion of oocysts from soil and feed, while rodents serve as intermediate hosts that perpetuate the parasite’s life cycle through predation by felids [38]. The presence of *T. gondii* in these species highlights the ecological complexity of transmission and the potential for cross-species spill over.

Collectively, these findings demonstrate the genetic and ecological diversity of *T. gondii* in Trishal and emphasize the zoonotic risk posed by contaminated environments and consumption of infected animal products. The detection of the parasite across multiple host species and sample types reinforces the need for integrated surveillance and control strategies, including public education on safe meat handling, improved livestock management, and targeted interventions to reduce feline shedding. Future studies should focus on genotyping the detected strains to elucidate lineage-specific transmission dynamics and assess their pathogenic potential in both animals and humans.

The multilocus genotyping of *T. gondii* isolates is characterized by the predominance of Type I and mixed genotypes (I/II, I/III) in cattle, cats, dogs, and rodents. These findings deviate from the classical clonal population structure observed in Europe and North America, where Type II strains dominate, and instead reflect the genetic heterogeneity increasingly reported in parts of Asia and South America [21,34]. The presence of Type I and mixed allelic patterns is particularly concerning, given their association with increased virulence and severe clinical outcomes in both animals and humans [29].

Cattle-derived isolates (Samples A–F), including those from Holstein Friesian and local breeds, predominantly exhibited Type I and mixed genotypes (I/III, I/II), aligning with reports from India and China where atypical or Type I strains were found in bovine tissues [15,39]. Although cattle are traditionally considered poor hosts [40], the detection of *T. gondii* DNA in fetal and placental tissues supports the possibility of vertical transmission [41].

Isolates from goats (Sample G) and sheep (Sample H) were primarily Type II or II/III, consistent with findings from Pakistan and Iran, where small ruminants frequently harbor non-clonal genotypes [42–44]. Although the type II and III genotypes, which are generally considered less virulent but still capable of causing reproductive losses and congenital infections [37].

The chicken isolate (Sample I) showed a mixed II/III profile, echoing previous studies that chickens, as ground-feeding animals, are highly susceptible to oocyst ingestion and serve as effective sentinels for environmental contamination [45,46]. The presence of Type I alleles in chicken brain tissue may indicate environmental contamination through oocyst shedding by cats, raising food safety concerns.

Fecal samples from cats (Sample J) and dogs (Sample K) revealed allelic combinations consistent with either mixed infections or potential recombinant strains, reflecting their role as definitive hosts [21], where sexual recombination of *T. gondii* occurs, generating novel genotypes that may be shed into the environment via oocysts [35]. However, as sequencing data were not available, these interpretations remain tentative and should be viewed as indicative rather than conclusive evidence of recombination. Dogs, while not definitive hosts, may act as mechanical carriers or acquire tissue cysts through scavenging, particularly near slaughter zones [47].

Rodent isolates (Sample L), collected near rice fields and slaughterhouses, exhibited Type I/II genotypes, similar to findings from Vietnam and Indonesia where rodents serve as reservoirs of virulent strains in rural ecosystems [38]. Their role in maintaining sylvatic transmission cycles alongside felids warrants further investigation.

The allelic patterns observed across species are consistent with either mixed infections involving multiple *T. gondii* lineages or potential recombinant strains circulating in the environment. The co-circulation of clonal (Type I) and non-clonal (Type II, III, and mixed) genotypes in a confined rural area suggests complex transmission dynamics. Because genotyping was limited to PCR-RFLP, we cannot conclusively differentiate mixed infections from recombination events. The detection of such genotypic diversity across multiple species further supports the hypothesis of environmental circulation of non-clonal strains and potential interspecies transmission. Such genetic heterogeneity may arise from repeated exposure to oocysts from different sources or from co-infections that facilitate recombination events in intermediate hosts [19]. The presence of virulent Type I strains in food animals such as cattle and goats pose a significant public health risk, particularly in communities where raw or undercooked meat is consumed.

The overall infection rate of 21.76% was observed, with the highest prevalence observed in cyst samples from slaughtered cattle (42.9%) and cat feces (33.3%). The detection of *T. gondii* DNA across diverse host species and sample types underscores the widespread presence and complex transmission dynamics of this parasite in the region. Importantly, the significant association between infection and consumption of surface water sources highlights a critical environmental risk factor. Poor slaughterhouse hygiene, improper disposal of infected tissues, and the presence of scavenging cats, dogs, and rodents further contribute to sustained transmission cycles. These findings emphasize the urgent need to improve water safety, farm biosecurity, and public awareness to reduce zoonotic risk. Future studies incorporating environmental sampling including water and soil and human health assessments are essential to fully elucidate transmission pathways and inform effective control strategies to mitigate the burden of toxoplasmosis in Bangladesh.

The detection of Type I and atypical *T. gondii* genotypes in food-producing animals and commensal species in Trishal Upazila has important implications for human health. Consumption of undercooked meat from infected cattle, goats, or sheep may expose local communities to virulent strains, while shared water sources, including ponds and rivers, represent a potential route of oocyst transmission to humans during bathing, washing, or drinking. Rural households frequently engage in close contact with livestock and free-ranging cats, increasing the risk of exposure, particularly among pregnant women and immunocompromised individuals. These findings underscore the need for targeted public health interventions, including improved farm hygiene, safe water management, proper meat handling, and community education to reduce zoonotic transmission of *T. gondii* in rural Bangladesh.

### Limitations

This study has several limitations that should be acknowledged. First, the purposive sampling strategy focused on high-exposure environments, such as smallholder farms and slaughterhouses, which may limit the generalizability of the prevalence estimates to the broader livestock population in Bangladesh. Second, only a limited number of isolates from each host species were successfully genotyped, restricting the ability to draw definitive conclusions regarding genotype distribution and interspecies transmission patterns. Third, although both univariate and multivariable logistic regression analyses were performed, the sample size constrained the statistical power to detect weaker associations, and residual confounding cannot be excluded. Finally, while the B1 gene-based nPCR assay used in this study is highly sensitive and specific, the possibility of cross-reactivity with closely related apicomplexan DNA cannot be entirely ruled out. While additional markers such as SAG1 or the 529 bp repeat element could enhance sensitivity and specificity, resource and sample constraints limited their inclusion in this study. Future studies incorporating random sampling, larger datasets, and sequencing-based genotyping would provide more comprehensive insights into the epidemiology and molecular diversity of *T. gondii* in Bangladesh.

## Conclusion

This study represents the first molecular genotyping of *Toxoplasma gondii* in animals from Bangladesh, revealing an overall infection rate of 21.76% across multiple host species. The detection of Type I and atypical genotypes (I/II, I/III, II/III) indicates the circulation of potentially virulent strains of *T. gondii* among cattle, goats, sheep, cats, dogs, chickens, and rodents in Trishal Upazila, Mymensingh district. The significant association between infection and the use of pond or river water as a drinking source highlights the importance of environmental contamination in parasite transmission. Although other factors such as poor carcass disposal, lack of rodent control, and unhygienic slaughter practices showed non-significant trends, they suggest a potential association with *T. gondii* infection. Overall, these findings provide baseline genetic and epidemiological data for *T. gondii* in Bangladesh and underscore the need for improved water hygiene, biosecurity, and further molecular studies to elucidate transmission dynamics and assess public health implications.

## Supporting information

S1 Raw Images Gel electrophoresis of PCR and multilocus PCR-RFLP products for *Toxoplasma gondii* detection and genotyping. (PDF)
