# Supplementary material for: Genotype distribution and risk factors of Toxoplasma gondii infection in animals of Trishal, Bangladesh
Source: PLoS One. 2026 Jan 13;21(1):e0340911. doi: 10.1371/journal.pone.0340911 (PMC12798977; doi:10.1371/journal.pone.0340911)
Supplement: S1 Raw Images — (PDF) [file pone.0340911.s001.pdf]

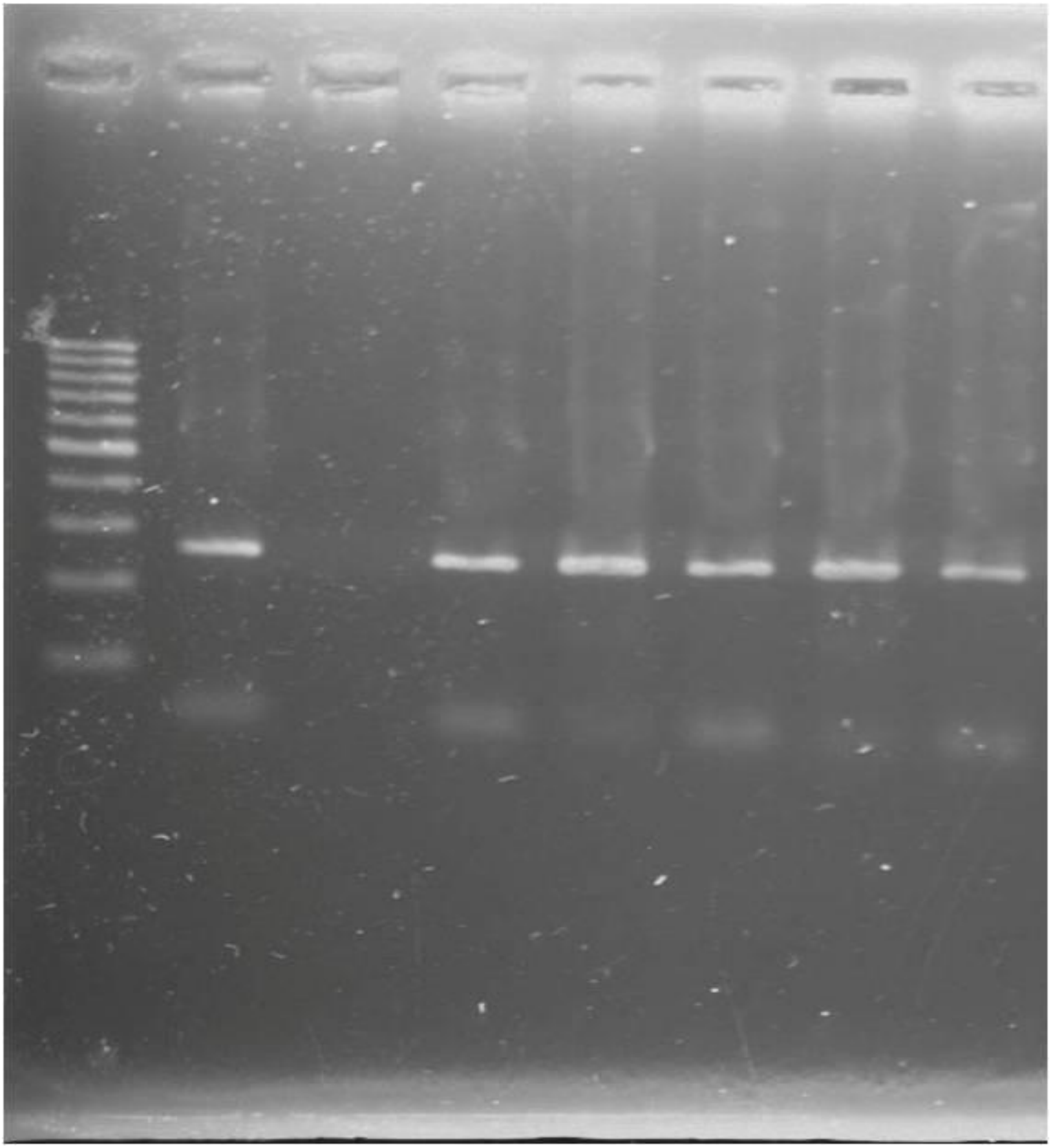

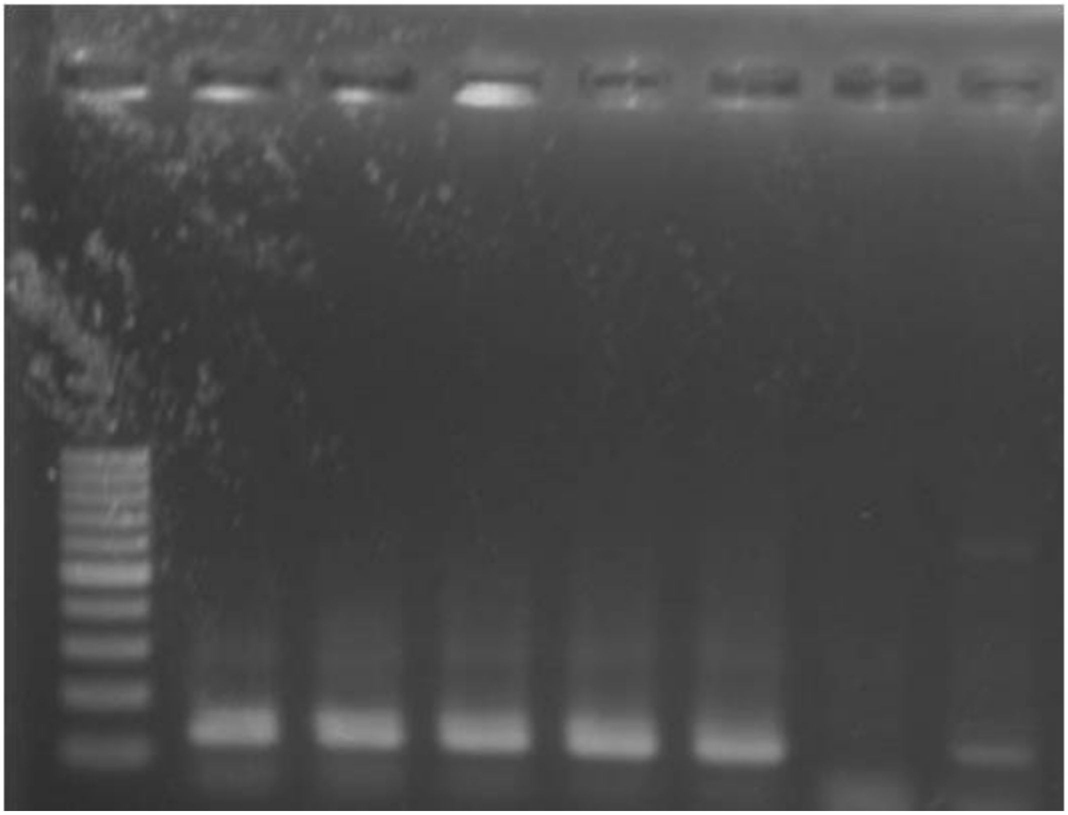

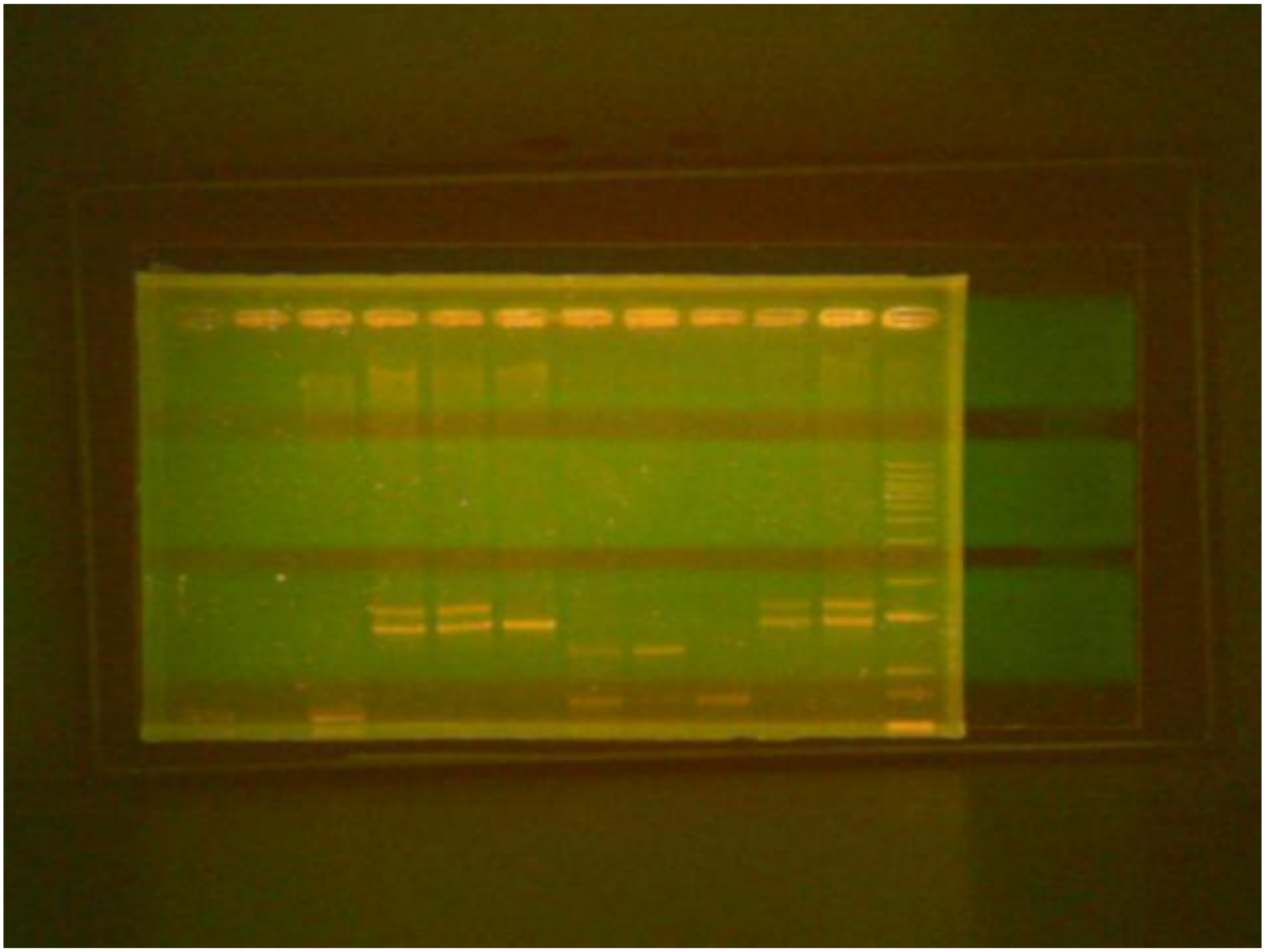

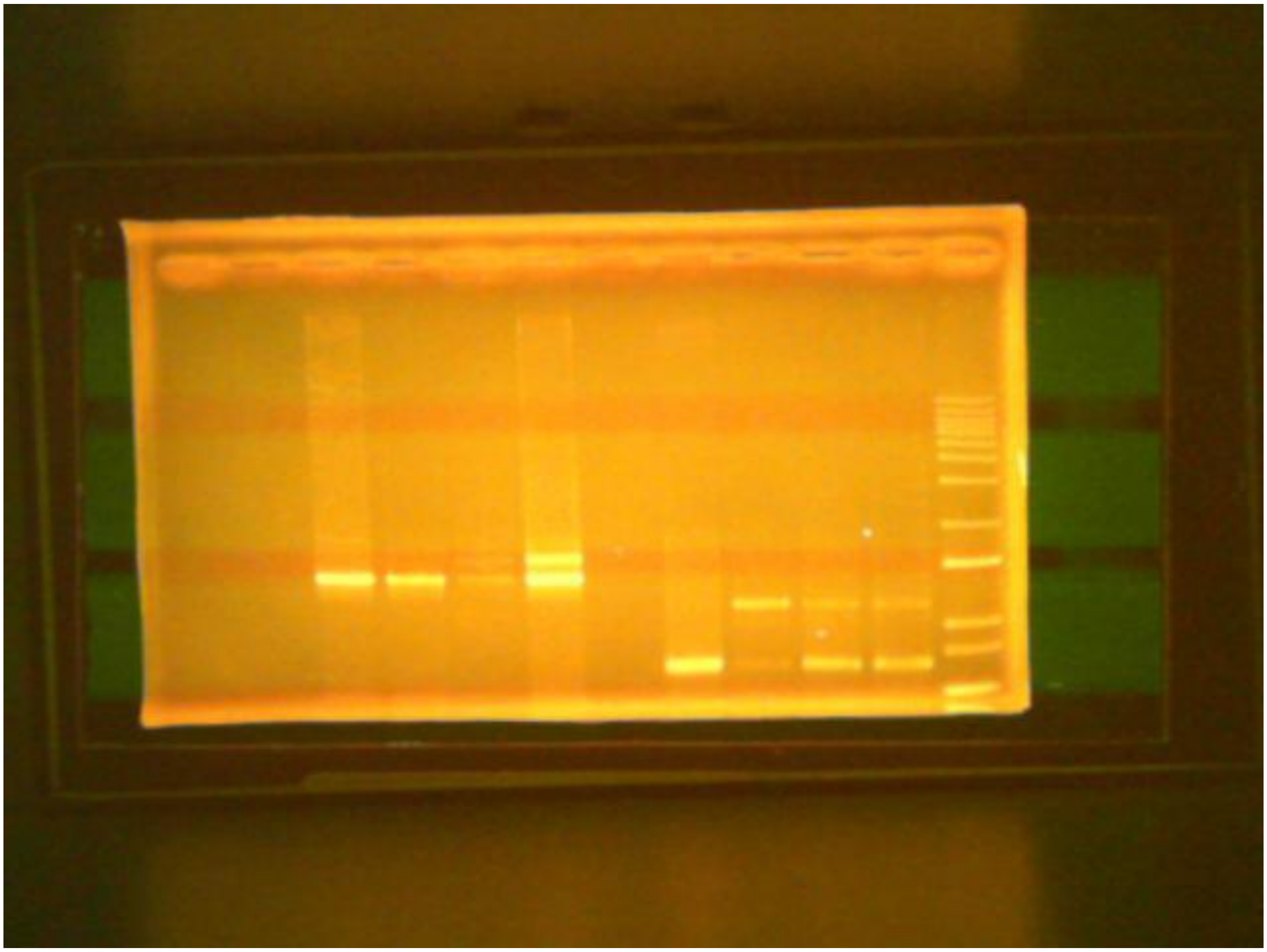

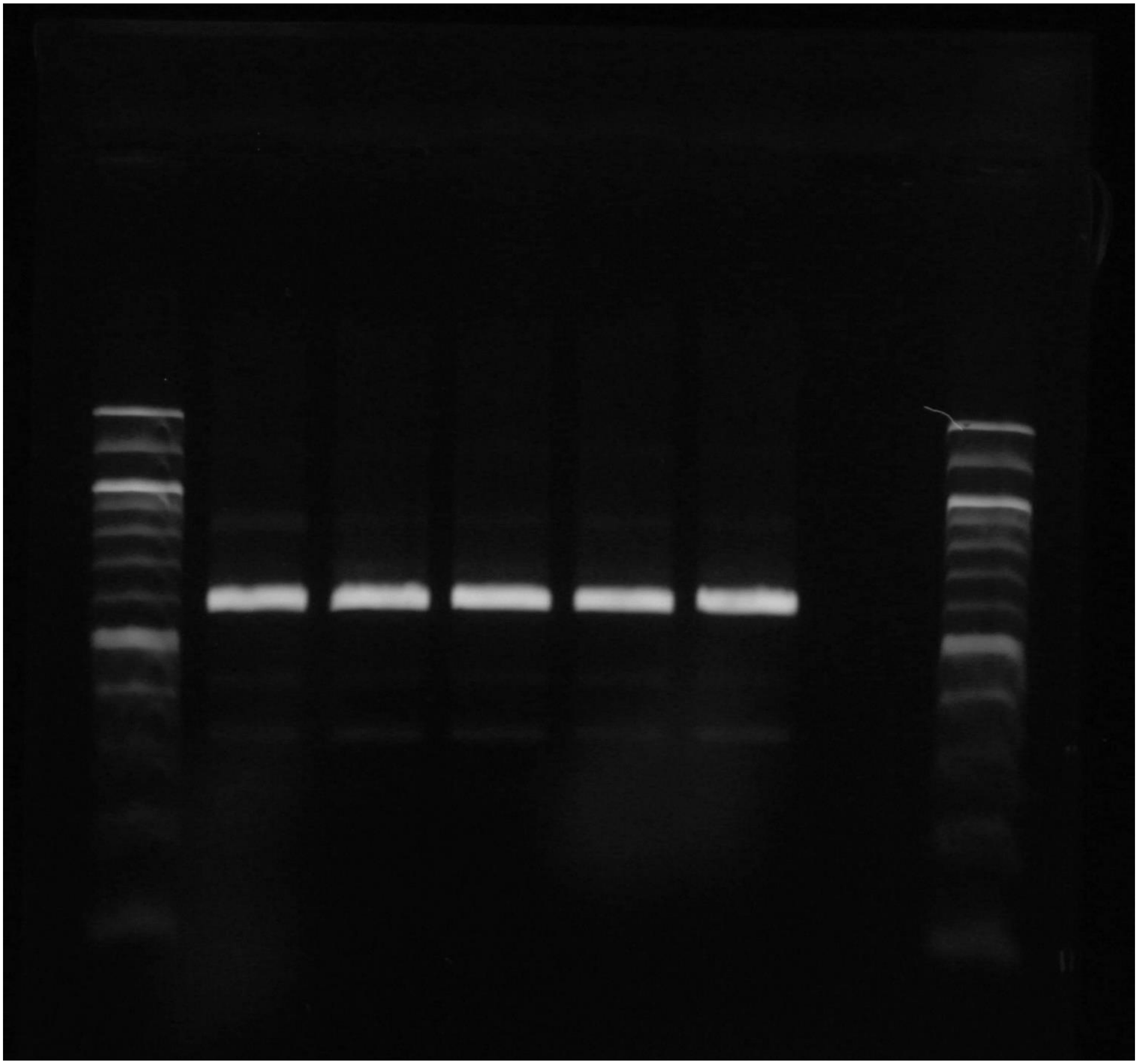

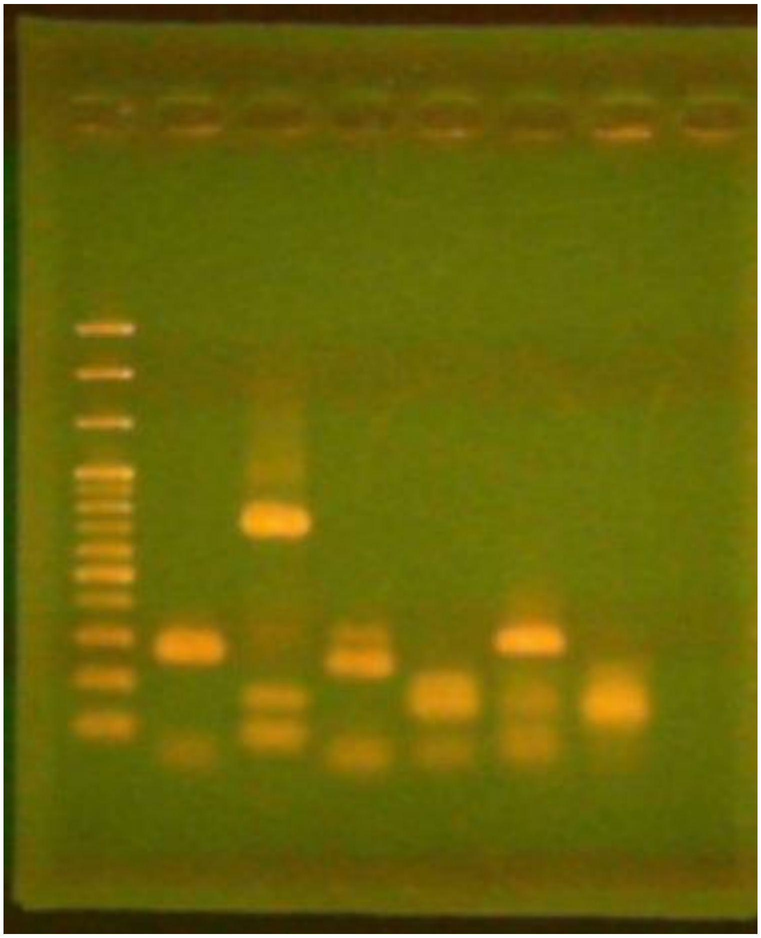

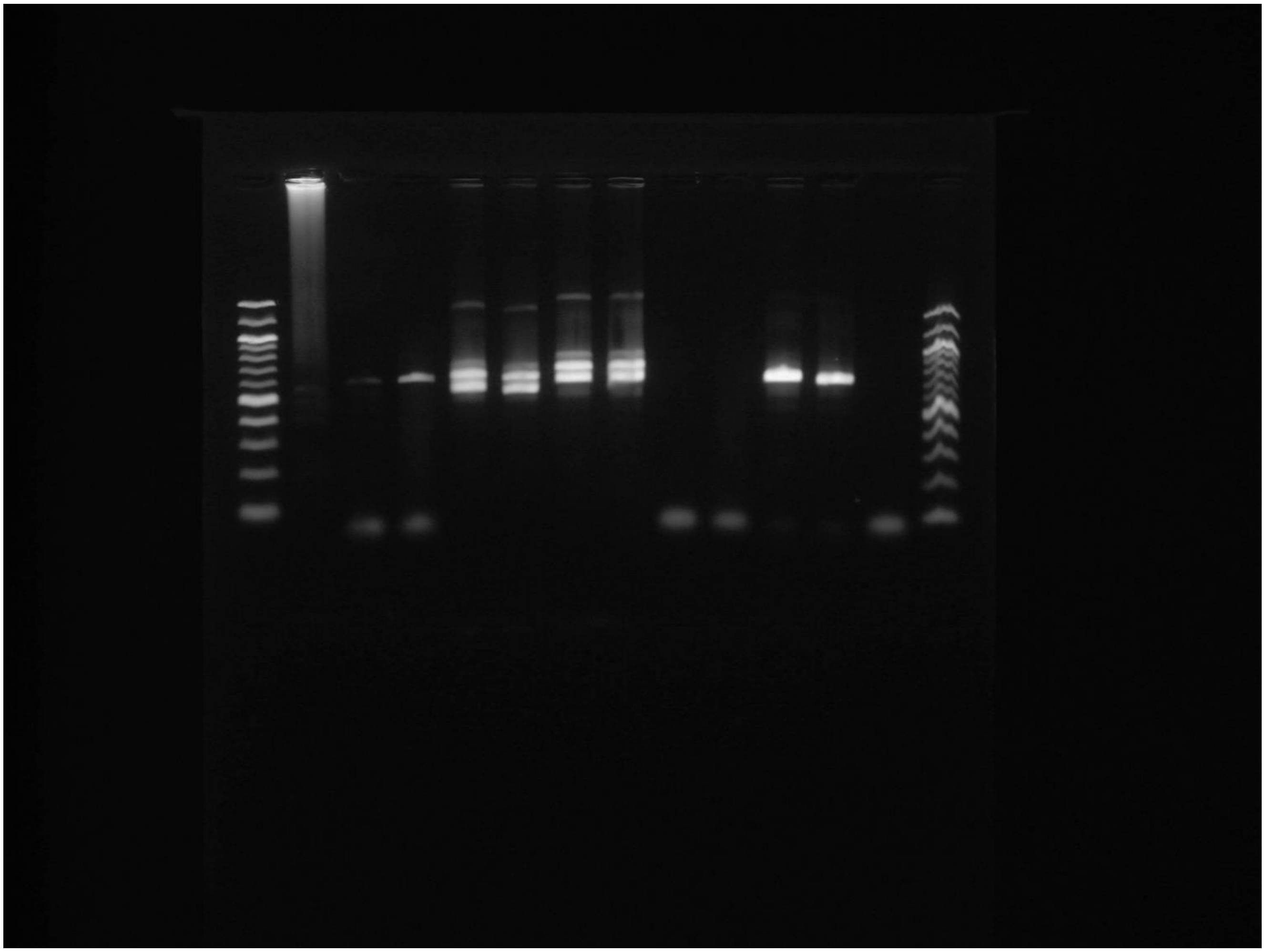

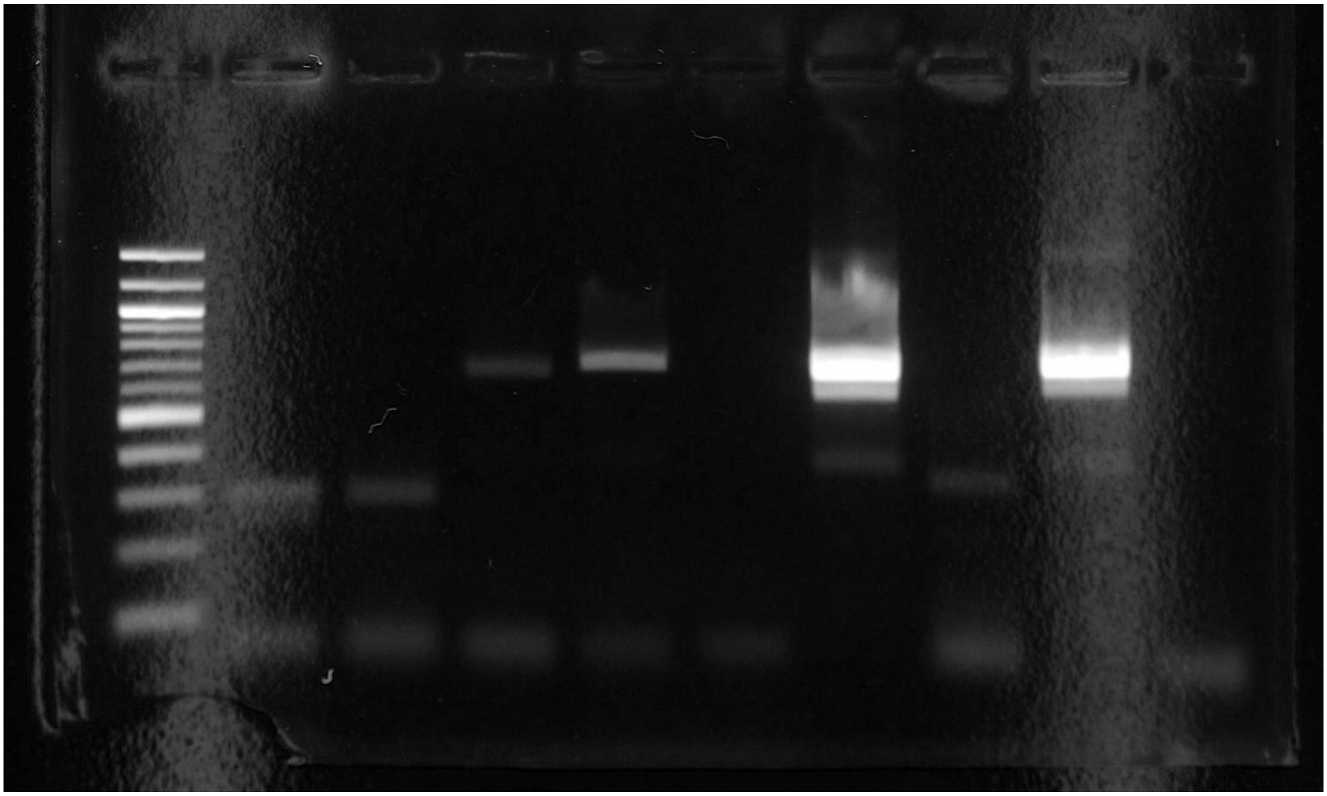

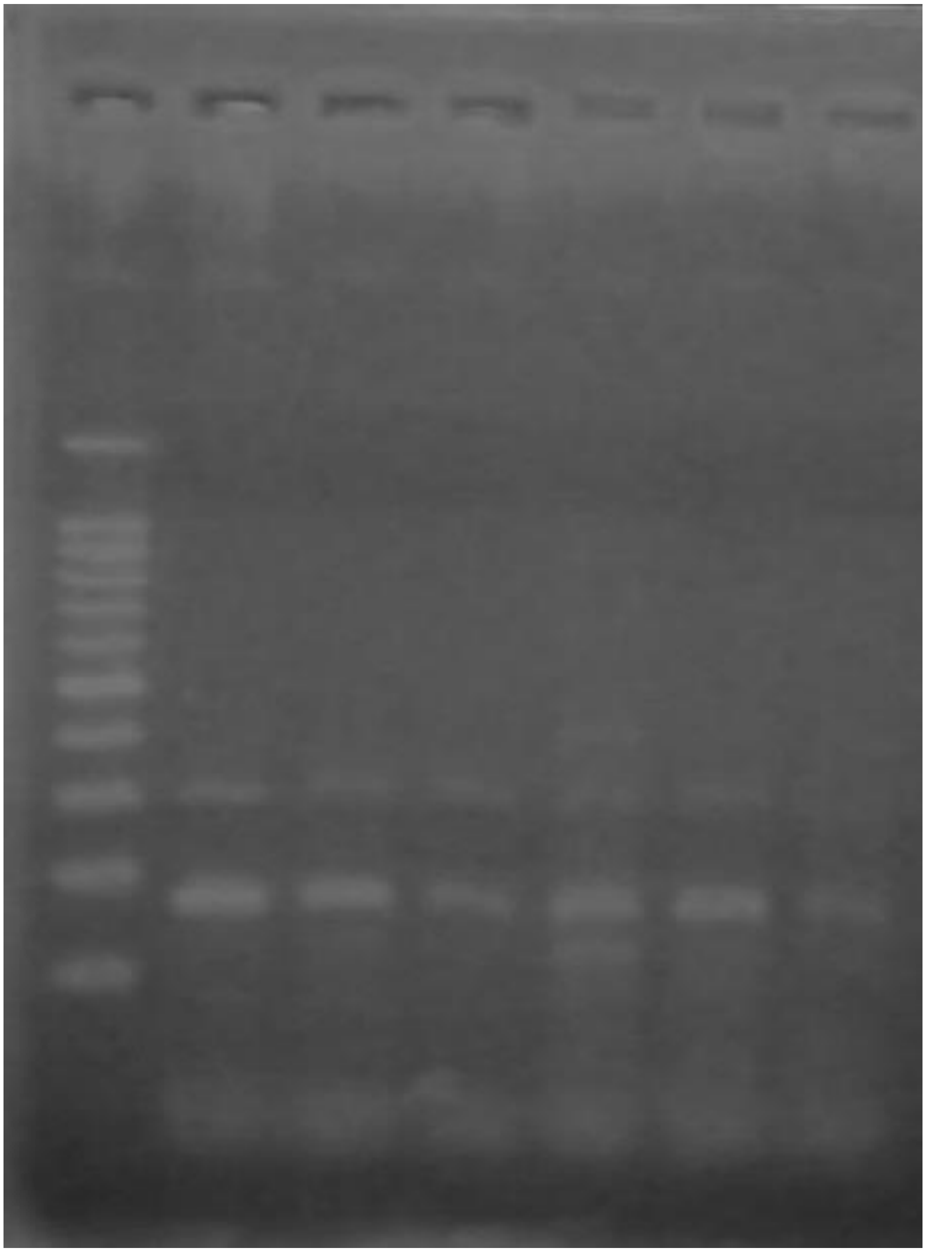

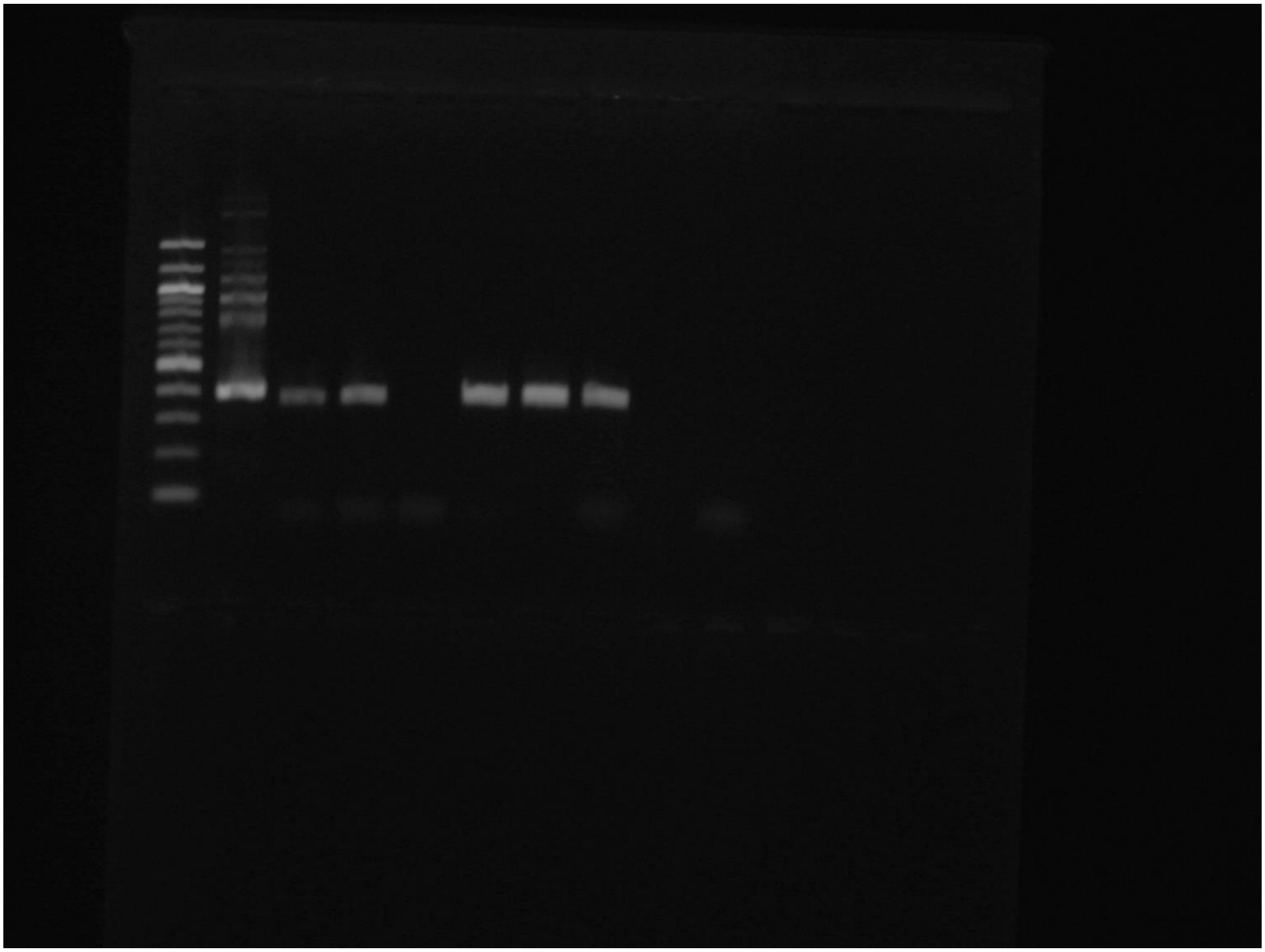

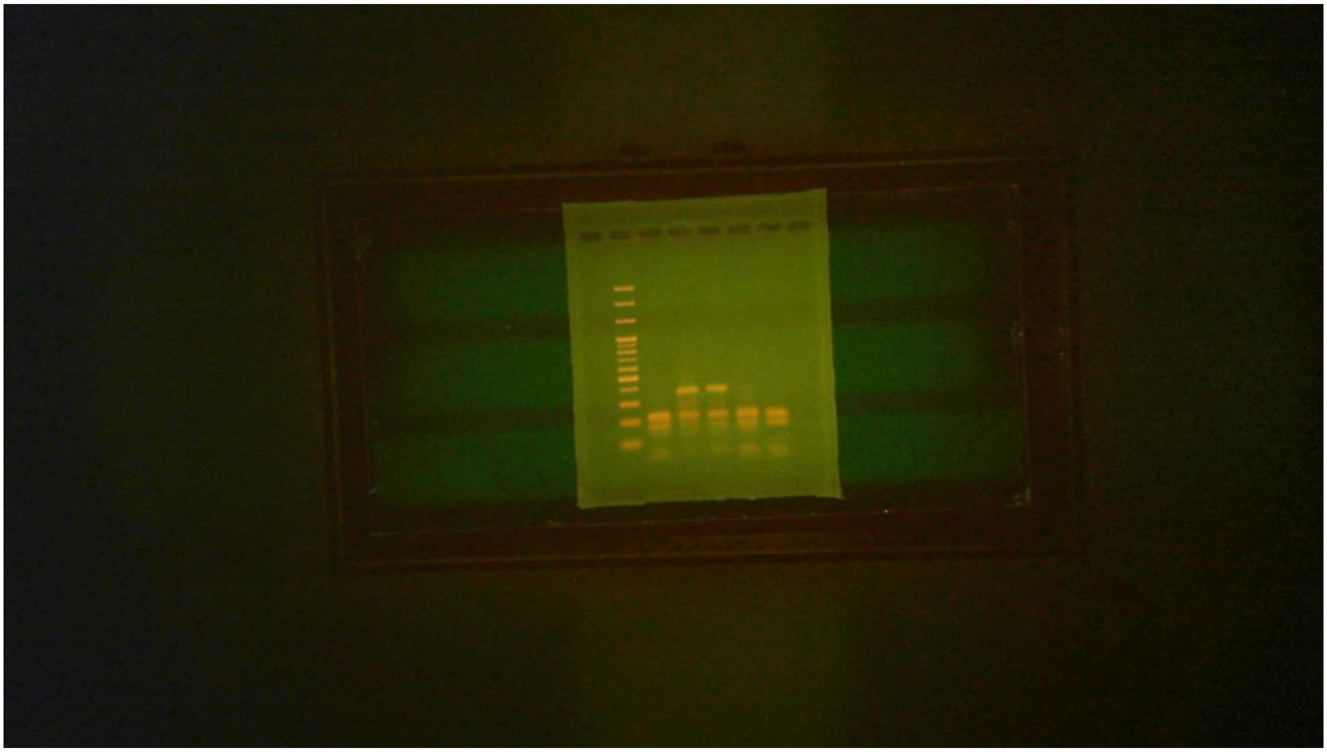

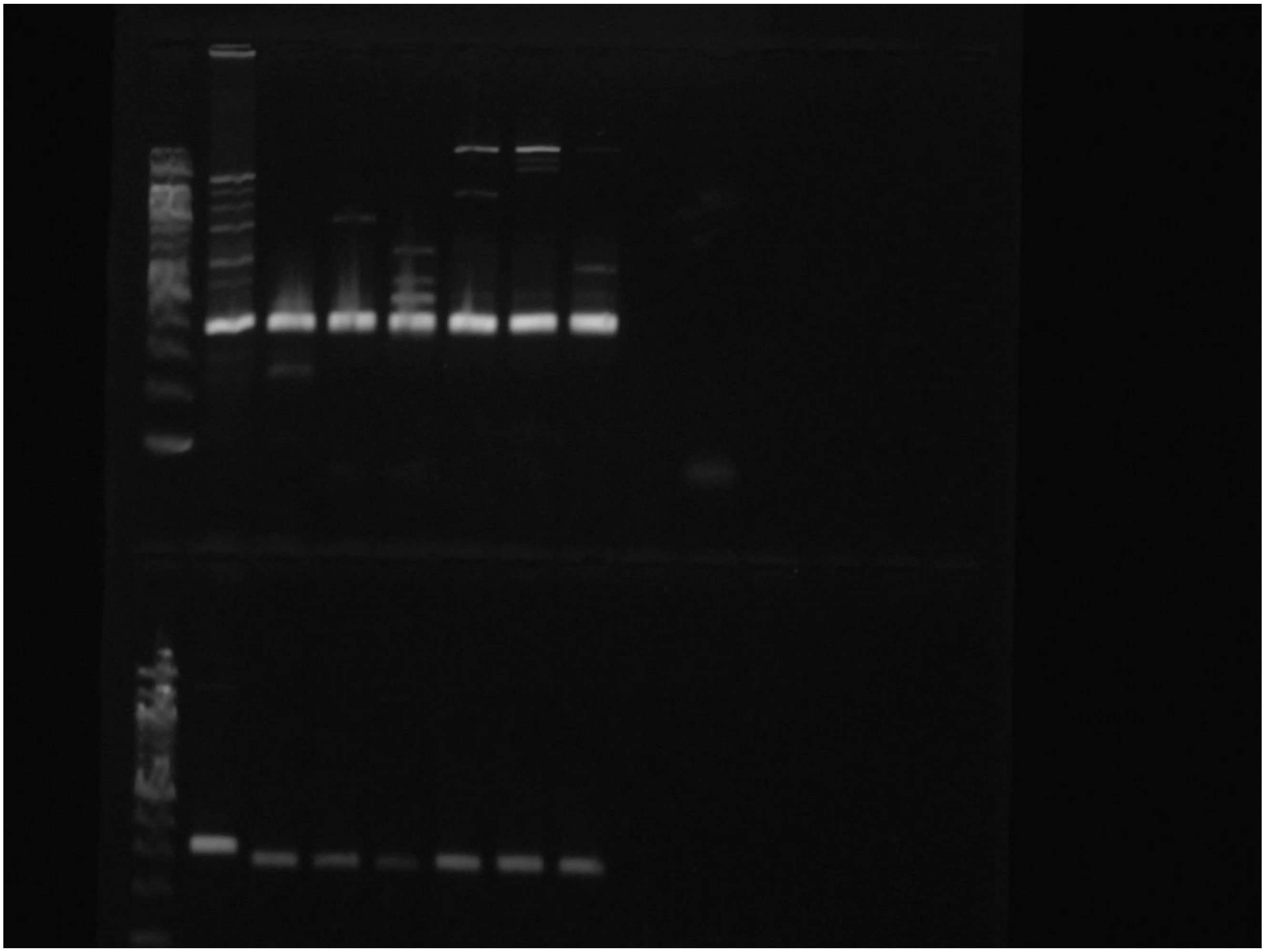

S1 Raw Images. Gel electrophoresis of PCR and multilocus PCR-RFLP products for *Toxoplasma* gondii detection and genotyping

This file contains raw gel electrophoresis images documenting molecular detection and genotyping of *Toxoplasma gondii* isolates.

- **Page 1:** Gel image showing amplification products from primary PCR targeting the *T. gondii* B1 gene (expected amplicon: 288 bp).
- **Page 2:** Gel image showing amplification products from nested PCR targeting the *T. gondii* B1 gene (expected amplicon: 114 bp).
- **Pages 3–12:** Gel images of multilocus nested PCR-RFLP analysis using the following genetic markers:
    - **Page 3:** SAG1
    - **Page 4:** alt. SAG2
    - **Page 5:** 5′SAG2
    - **Page 6:** 3′SAG2
    - **Page 7:** SAG3
    - **Page 8:** PK1
    - **Page 9:** BTUB
    - **Page 10:** GRA6
    - **Page 11:** C22-8
    - **Page 12:** C29-2

Each gel includes representative sample lanes, molecular weight markers, and controls. Band patterns were used to assign multilocus genotypes for *T. gondii* isolates.
